# OpenRad: a curated repository of open-access AI models for radiology

**DOI:** 10.1007/s00330-026-12650-0

**Published:** 2026-05-28

**Authors:** Konstantinos Vrettos, Galini Papadaki, Emmanouil Brilakis, Matthaios Triantafyllou, Dimitrios Leventis, Despina Staraki, Maria Mavroforou, Eleftherios Tzanis, Konstantina Giouroukou, Michail E. Klontzas

**Affiliations:** 1 Artificial Intelligence and Translational Imaging (ATI) Lab, Department of Radiology, School of Medicine, University of Crete, Heraklion, Greece; 2 Division of Radiology, Department of Clinical Science Intervention and Technology (CLINTEC), Karolinska Institute, Huddinge, Sweden; 3 Computational Biomedicine Laboratory, Institute of Computer Science - Foundation for Research and Technology Hellas (ICS-FORTH), Heraklion, Greece

**Keywords:** Artificial intelligence, Model, Radiology, Open-access, Repository

## Abstract

**Objectives:**

To create and evaluate OpenRad (https://konstvr.github.io/OpenRad/index.html), a curated, standardized repository that aggregates open-access radiology artificial intelligence (AI) models enriched with metadata from the corresponding code repositories regarding availability of pretrained weights and interactive applications.

**Materials and methods:**

Retrospective analysis of literature from PubMed, arXiv, and Scopus until 12/2025 (5239 works). After duplicate removal and relevance screening, 1694 articles describing open-access AI models were processed. Model records were generated using a locally hosted large language model (LLM) (gpt-oss:120b), based on the RSNA AI Roadmap JSON schema, and then manually verified by ten expert reviewers. The stability of LLM outputs was assessed on 225 randomly selected papers using text similarity metrics. A statistical analysis of the collected works was also performed.

**Results:**

The included 1694 models span all imaging modalities (computed tomography (CT), magnetic resonance imaging (MRI), X-ray, ultrasound (US)) and radiology subspecialties. Automated extraction demonstrated high stability for structured fields (Levenshtein ratio > 90%), with 78.5% of edits, during expert review, being minor corrections. Statistical analysis of the repository revealed convolutional neural network (CNN) and transformer architectures as dominant, while MRI was the most commonly used modality (in 621 neuroradiology AI models). Research output was mostly concentrated in China and the United States. The proposed web interface enables model discovery via keyword search and filters for modality, subspecialty, intended use and demo availability, alongside live statistical dashboards. The community can also contribute new models through a dedicated portal.

**Conclusion:**

OpenRad contains ~1700 open-access, curated radiology AI models with standardized metadata, supplemented with analysis of code repositories, thereby creating a comprehensive, searchable resource for the radiology community.

**Key Points:**

***Question*** *Current repositories of AI models in radiology are limited, fragmented and include models that are not readily available for use*.

***Findings*** *OpenRad, a curated repository, includes ~1700 open-access, standardized radiology AI models with verified code repositories*.

***Clinical relevance*** *OpenRad enables the radiology community to reliably access AI models with readily available code, weights and demos*.

**Graphical Abstract:**

**Figure:**
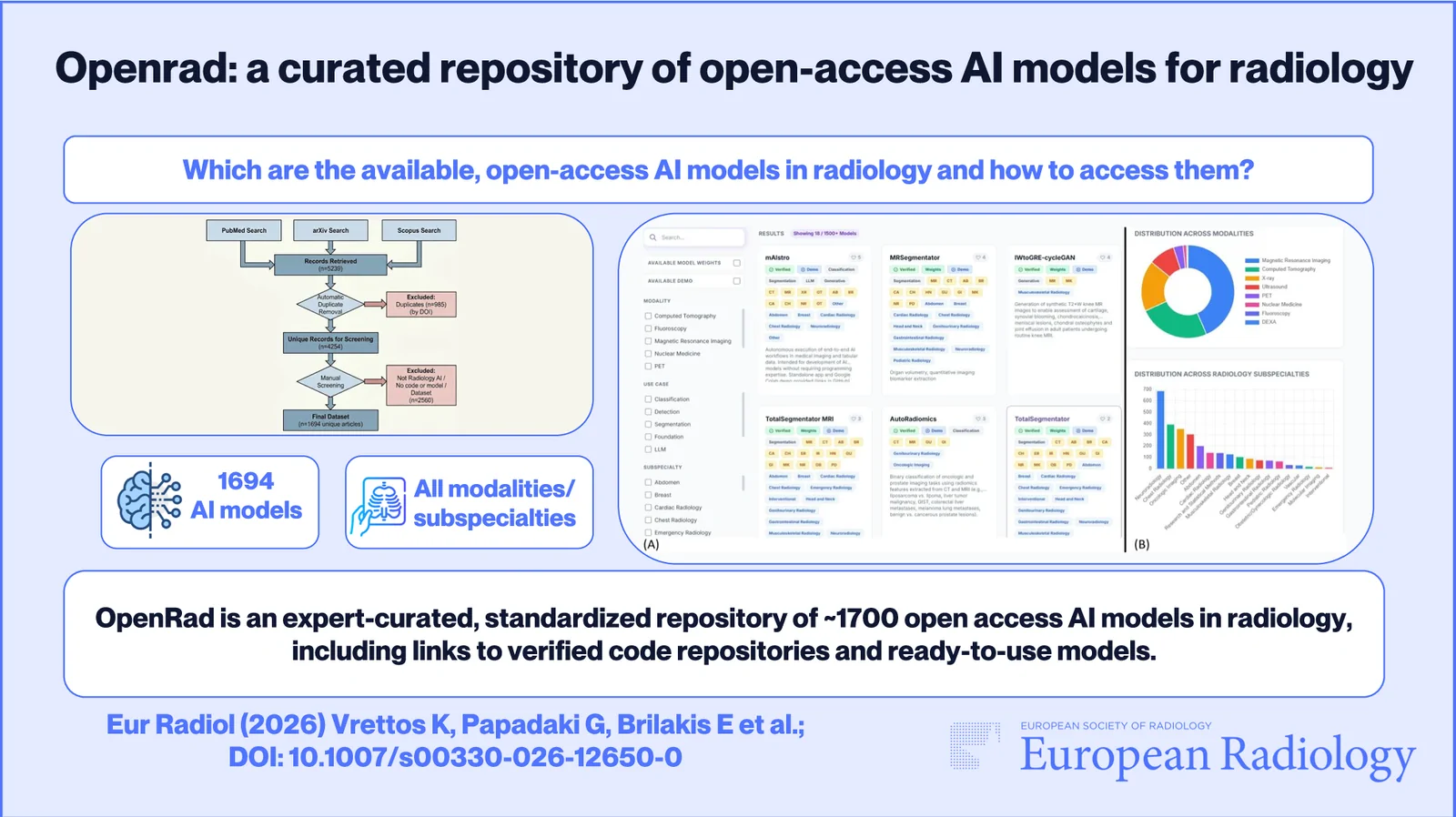

## Introduction

In recent years, the radiology community has witnessed an unprecedented surge of artificial intelligence (AI) research [1]. Numerous deep-learning models for report writing [2], lesion detection [3], segmentation [4] and many other tasks have been reported in conferences, journals and pre-print servers. Yet, the very abundance of these methods has created a new bottleneck: discoverability, reproducibility and clinical translation are hampered by a fragmented landscape of model distribution.

Published models are scattered across a variety of sources, including supplementary material, personal GitHub repositories, institutional webpages, or proprietary platforms, forcing users to conduct exhaustive manual searches to locate a model that matches their specific requirement. In addition, the lack of standardized metadata makes it challenging to assess whether a model is appropriate for a given clinical setting or to compare it fairly against alternative solutions [5]. The first step to address this issue was the introduction of reporting guidelines for papers relevant to AI models in radiology [6, 7]. Expanding on this approach, the Radiological Society of North America (RSNA) proposed the AI Roadmap, which suggests standardized ontology terms (RadLex) and format, for reporting AI model data in radiology that disclose details such as intended use, model structure and licensing information [8, 9, 10]. Nonetheless, adherence to these standards is not widespread, which limits the availability of structured, machine-readable model records. Existing AI model repositories [11–15] include a relatively limited, handpicked number of model cards, some of which refer to datasets without accompanying models. Importantly, they include manuscripts without open access code that are not reproducible and may not be useful to researchers attempting to develop new models or to apply existing models to enhance radiology workflows.

The aim of this work was to create OpenRad, a comprehensive, up-to-date, searchable public repository of open-access radiology AI models, that provide open-access code or models for public use. The proposed work offers: (i) an expert-curated collection of 1694 models spanning all imaging modalities and radiology subspecialties, (ii) standardized model records using the ROADMAP ontology (RadLex terms), ensuring consistent reporting, (iii) additional information derived from the analysis of code repositories, such as the availability of trained models or interactive implementations, (iv) option for users to submit new models following the standardized format, (v) live statistical data, based on the included models, capturing the current state of open-access AI research in radiology. This online repository enables model discovery for researchers, clinicians and industry partners, facilitating research use and the translation of promising algorithms into clinically useful tools.

## Materials and methods

Institutional Review Board (IRB) approval was not required for this study as it was limited to the analysis of publicly available literature and existing AI models. This work did not involve human subjects.

### Initial dataset creation

**Fig. 1:**
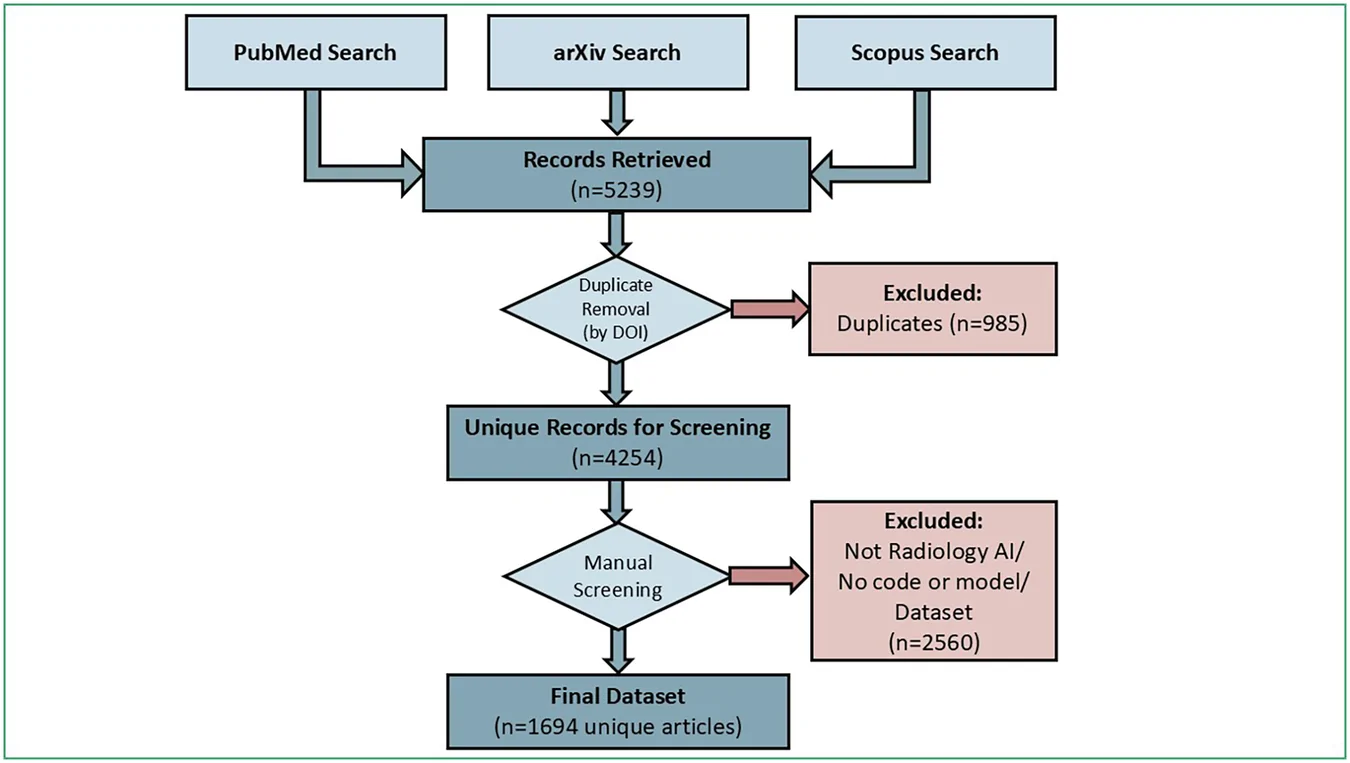
Flow diagram of the article selection process for the construction of the OpenRad repository

A comprehensive corpus of radiology AI papers was formed by scraping PubMed, arXiv and Scopus using the queries in Supplementary Table 1 and including works published until 12/2025. This search retrieved 5239 records that span medical literature, but also computer science journals and conference proceedings, which carry a large volume of AI models. Duplicates across databases were removed using the digital object identifier (DOI) (985 duplicates), and manual screening excluded papers that were not focused on a radiology AI model (1832 papers). Importantly, papers that did not provide open access code/model (704) or referred to datasets (24) were subsequently excluded, yielding a final dataset of 1694 unique articles (Fig. 1), where all presented models are accompanied by ready-to-use code/model.

To transform this corpus into a standardized collection of models, the RSNA AI Roadmap reference schema (https://github.com/RSNA/ATLAS/blob/main/model.json) was followed. To construct the standardized model records, we developed an automated pipeline using the open-source large language model (LLM) gpt-oss:120b (run locally), which extracted structured data from the included works. Python 3.10 with the “instructor” library was used to issue structured prompts that populated the selected fields of the RSNA JavaScript Object Notation (JSON) schema. When a full‑text portable document format was available, it was supplied to the LLM, otherwise the abstract was used. In addition to the recommended RSNA fields (modality, clinical subspecialty, intended use, architecture, performance metrics), the template was enriched with attributes regarding dataset details and validation strategy. The generated model records also include an assessment of the contents of the GitHub repository, if one is provided. This was carried out by automatically querying all URLs identified as code repositories and identifying the presence of pretrained weights or ready-to-use implementations (demos) of the models. The final JSON files contained bibliographic metadata and the extracted technical details.

### LLM‑generated model record assessment: stability and ground‑truth comparison

To evaluate the reliability of the automatically generated records, the intra‑LLM stability was checked. A subset of 225 articles was repeatedly processed, with the first generation serving as the reference. The stability was evaluated across key fields such as title, author list, affiliations, repository URL, sustainability (training time/hardware), architecture and regulatory details using three complementary similarity measures: Python’s “difflib.SequenceMatcher” (v3.14.2) (longest common subsequence ratio), Levenshtein ratio (character‑level edit distance), and Jaccard similarity (token‑set overlap).

### Expert curation of the OpenRad repository

Generated model records have been manually verified by 10 expert reviewers (three PhD candidates, 6 MSc candidates and 1 assistant professor, all working on AI research) to ensure correctness and avoid hallucinations. The reviewers assessed whether the extracted information faithfully reflected the source article data and made any necessary corrections, marking them as minor if they involved small inaccuracies like typos or incomplete metrics, or major for critical errors like missing fields or broken links.

### Descriptive analysis of the curated repository

The final curated set of JSON files was analyzed to reveal underlying trends. This included summarizing the most popular model architectures and validation strategies with bar charts using Seaborn (v0.13.2). Geospatial analysis was also performed by parsing author affiliations. ISO-3 country codes were mapped to organization names using a comprehensive dictionary of over 100 country names, major cities and research institutions. This data was visualized using choropleth maps generated with Plotly (v5.15.0)

Statistical analysis and visualizations were generated using a suite of Python plotting libraries. Matplotlib (v3.7.1) and Seaborn were utilized to create static visualizations, including bar charts for architecture frequency and heatmaps illustrating the cross-distribution of clinical specialties vs imaging modalities. Qualitative insights into model limitations were synthesized using the WordCloud library (v1.9.6) to identify dominant keywords in the reported limitation sections. Furthermore, a heatmap correlating the reported metrics to model type, such as classification or segmentation, was constructed.

## Results

### Platform overview

#### Open-access model discovery and filtering

The main OpenRad dashboard serves as the central hub for model discovery (https://konstvr.github.io/OpenRad/index.html). Users can search for models using keywords or refine their results through a comprehensive set of filters located in the sidebar. These filters include:

- Resource availability: finds models for which repositories are available (GitHub or other), providing open weights or trained models. This data has been collected by reviewing the repositories of all papers, in addition to the publication itself.
- Categorization: models are tagged by Modality (e.g., computed tomography (CT), magnetic resonance imaging (MRI), X-ray), Subspecialty (e.g., neuroradiology, chest radiology, musculoskeletal) and Use Case (e.g., classification, segmentation, detection). RadLex codes have been used for encoding modality and subspecialty, following the RSNA roadmap recommendations.
- Demo availability: filter for works that include interactive/downloadable applications.

**Fig. 2:**
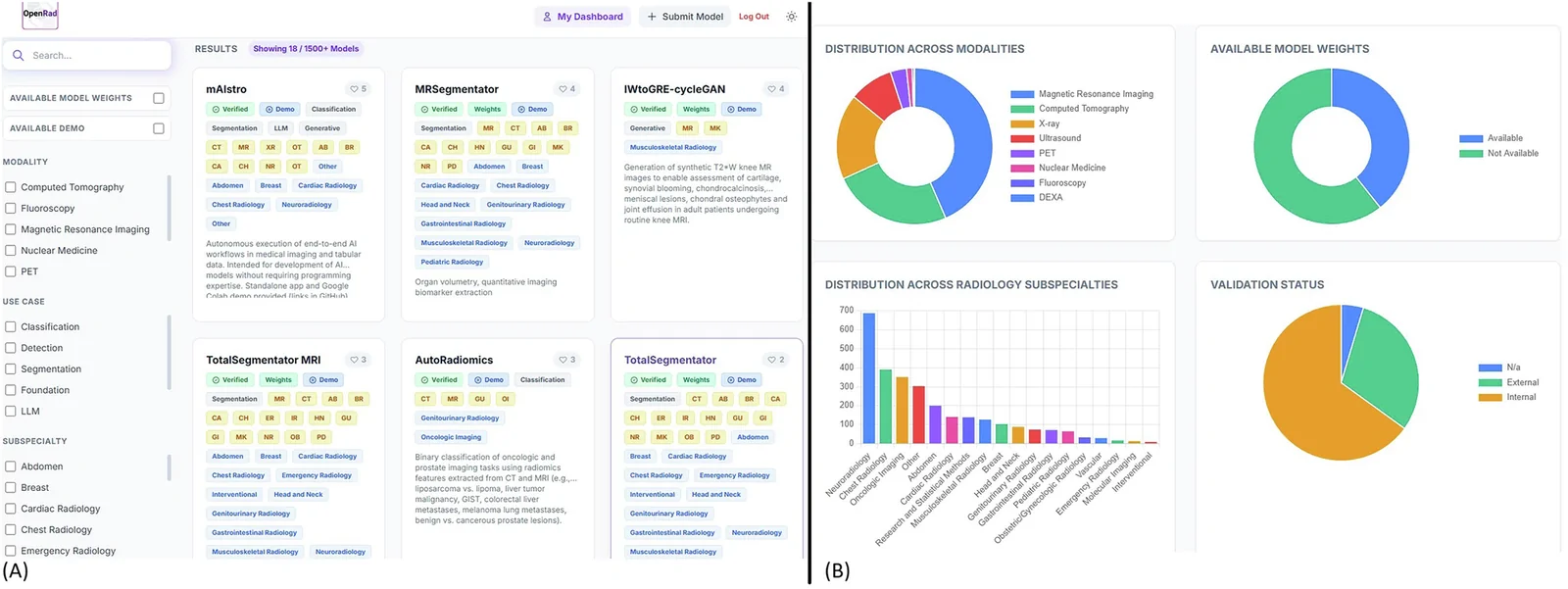
**A** OpenRad interface. **B** Live, interactive statistics based on the entire model collection

The search results are displayed as a responsive grid of objects (Fig. 2A), each providing a quick summary of the model, including its name and visual badges indicating its verification status, modality, subspecialty and availability of saved weights. This page also includes live and interactive overall statistics, regarding the included works, thus summarizing the current landscape of open access AI research (Fig. 2B).

#### Model record details

Clicking on a model navigates to the detailed view, which presents a structured and comprehensive overview of the model. The interface displays general information, such as the model’s name and architecture, alongside technical details regarding the used dataset(s). To assist users in evaluating model suitability, the view includes a section for reported performance metrics and known limitations. Finally, the page serves as a gateway to external resources, providing direct links to code repositories (such as GitHub), the original paper/abstract, and hosted interactive demos, when available. For the subset of models (27) with existing ATLAS model cards, that met our inclusion criteria, a link to ATLAS was also included in our model record.

#### Community contribution and curation

OpenRad operates on a community-driven model aimed at keeping the registry up-to-date and accurate. Users are encouraged to submit new models to the platform, in addition to the updates done by the authors, ensuring the database continuously expands with the latest research. All community submissions will be verified by experts before being included in the repository, to ensure the validity of submitted data and the consistency of the repository. Beyond submissions, the platform empowers registered users to act as curators. Through a “Verify & Edit” mode, contributors can suggest corrections to metadata or append missing information. These suggestions are tracked and subject to verification to maintain high data quality. Additionally, a flagging system facilitates community moderation, allowing users to report any issues.

### Model card assessment: intra‑LLM stability and human review

The stability analysis demonstrated an adequate level of consistency across duplicate models for the extracted metadata fields, based on lexical scores (Table 1). Structured content fields related to model identification including authors, affiliation list, repository link, title and sustainability, showed high stability. Complex, free-text fields such as model architecture, limitations and regulatory details exhibited higher variance, reflecting the model’s tendency to rephrase descriptive content between generations. Aggregate statistics (median and IQR) for each metric are reported in Table 1. The qualitative human review confirmed that despite these lexical differences, the technical descriptions exhibited high semantic consistency, indicating that the LLM successfully extracted and synthesized the core meaning of the text. Moreover, 78.5% of corrections made during the manual verification of 1694 included models, were classified as minor.

**Table 1:** Median lexical similarity scores and the respective IQR for fields in model records

Field name	Difflib	Levenshtein Ratio	Jaccard Similarity
Authors	100.0% (100%-100%)	100.0% (100%-100%)	100.0% (100%-100%)
Repository link	100.0% (100%-100%)	100.0% (100%-100%)	100.0% (100%-100%)
Title	100.0% (100%-100%)	100.0% (100%-100%)	100.0% (100%-100%)
Affiliation list	100.0% (100%-100%)	100.0% (100%-100%)	100.0% (100%-100%)
Sustainability (training time / Hardware)	100.0% (100%-100%)	100.0% (100%-100%)	100.0% (100%-100%)
Architecture	81.4% (52%–100%)	73.8% (40%–100%)	62.0% (29%–100%)
Limitations	36.9% (0%–100%)	41.3% (0%–100%)	29.5% (0%–100%)
Regulatory	100.0% (53%–100%)	100.0% (50%–100%)	100.0% (17%–100%)

*IQR* Interquartile Range

### Analysis of repository data

**Fig. 3:**
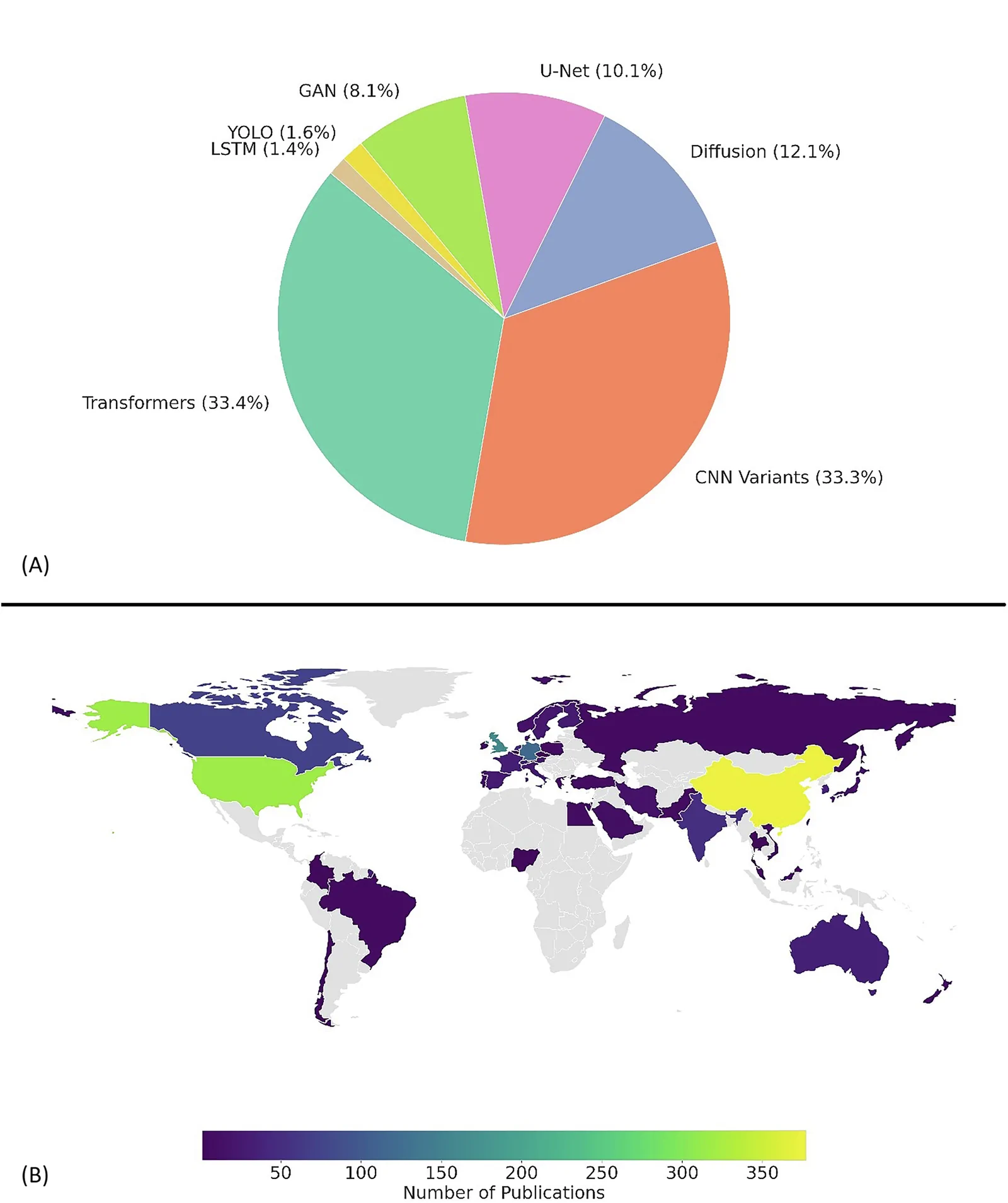
**A** Most frequent model architectures. **B** Global distribution of publications with open access AI models

**Fig. 4:**
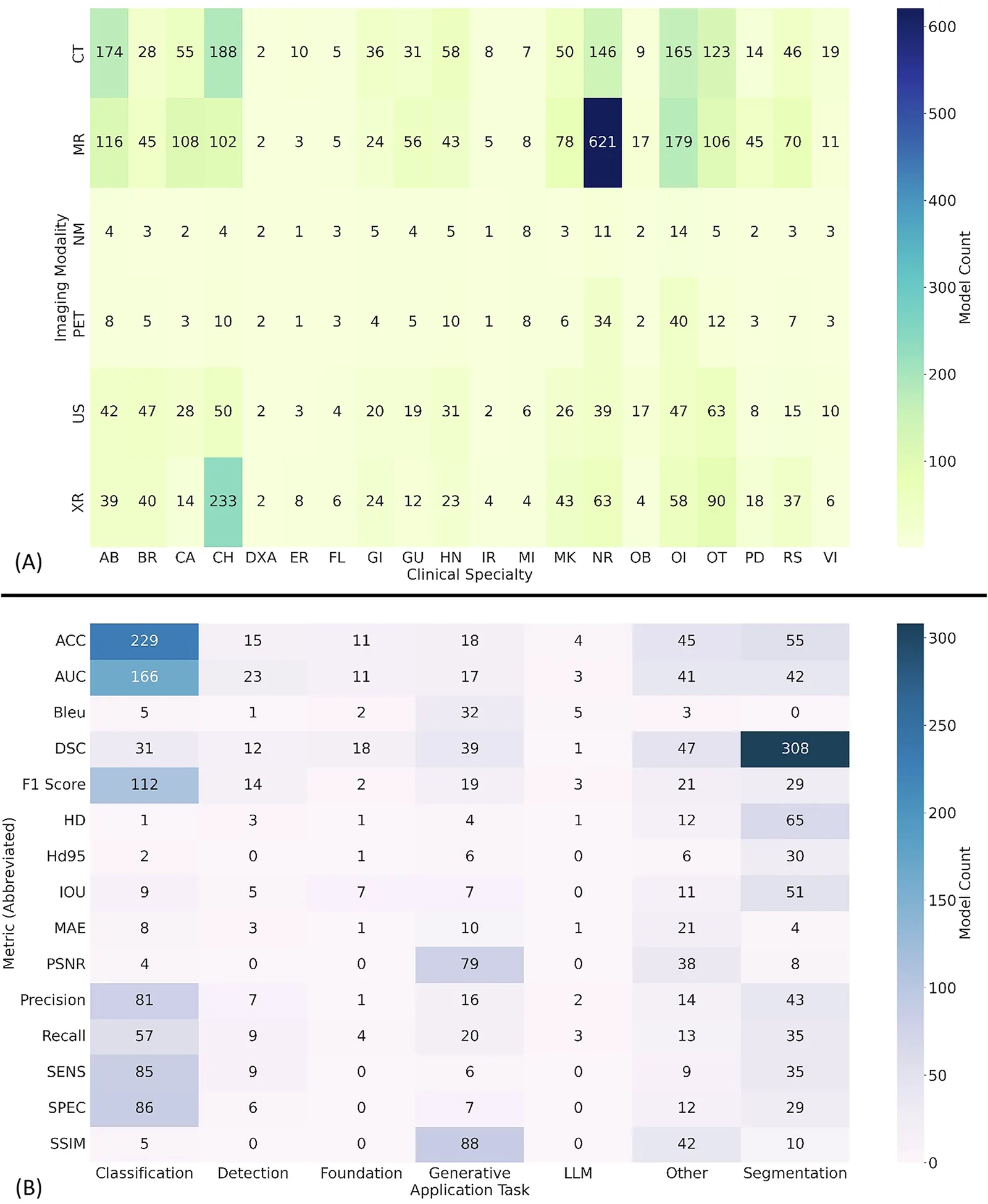
**A** Distribution of imaging modalities used by AI models across various radiology domains. AB, abdominal radiology; BR, breast radiology; CA, cardiac radiology; CH, chest radiology; ER, emergency radiology; FL, fluoroscopy; GI, gastrointestinal radiology; GU, genitourinary radiology; HN, head and neck; IR, interventional radiology; MI, molecular imaging; MK, musculoskeletal radiology; NR, neuroradiology; OB, obstetric/gynecologic radiology; OI, oncological imaging; OT, other; PD, pediatric radiology; RS, research and statistical methods; VI, vascular imaging. **B** Distribution of metrics used in the assessment of different model types. ACC, accuracy; AUC, area under the receiver operating characteristic curve; DSC, dice similarity coefficient; HD, Hausdorff distance; IOU, intersection over union; MAE, mean absolute error; PCC, Pearson correlation coefficient; PSNR, peak signal-to-noise ratio; SENS, sensitivity; SPEC, specificity; SSIM, structural similarity index measure

The analyses shown in Figs. 3 and 4 collectively characterize the current landscape of open-access AI models in radiology. Figure 3A displays the distribution of deep-learning architectures: variants of CNN and transformer-based architectures dominate (> 33%). Diffusion models are also frequently used (12.1%), followed by U-Net variants and generative adversarial networks. A smaller number of works utilized You Only Look Once (YOLO) models. Figure 3B illustrates the geographic concentration of publications, with China (≈ 400 papers) and the United States (≈ 300 papers) accounting for the majority of the output. Secondary contributions (50–150 papers) came from a limited set of European, Indian and East-Asian nations, while most remaining countries contributed only a handful of studies. Figure 4B presents metric co-occurrence by task type: classification metrics were led by accuracy (229), area under the curve (AUC) (166), F1-score (112) and specificity (86). Segmentation accuracy was most commonly measured with the Dice similarity coefficient (DSC) (308, which is ≈ 68% of total DSC mentions), followed by Hausdorff Distance (65) and accuracy (55). Generative tasks primarily used the structural similarity index measure (SSIM) (88) and PSNR (79). Figure 4A summarizes imaging modality-specialty cooccurrence, highlighting MRI as the most commonly used modality for neuroradiology applications (621 studies), with the next highest volumes observed for X-ray (233) and CT (188) for AI models focusing on chest. Regarding models for Oncological imaging (OI), MRI (179) and CT (165) were the most utilized modalities. A word-frequency analysis of reported limitations showed that “limited” was the most common term, followed by “data, model, dataset, training, performance, external validation” (Supplementary Fig. 1).

## Discussion

This study introduces the largest publicly accessible, curated repository that aggregates 1694 works on radiology‑AI models. By coupling bibliographic metadata with a systematic inspection of the linked code repositories, the platform supplies not only the descriptive information traditionally reported in other databases and research manuscripts, but also an objective appraisal of whether code, pretrained weights, or ready‑to‑use demos are actually available. This dual‑layer documentation has the potential to accelerate the transition from literature review to AI implementation by retrieving code, saved weights, or trained models, and, in many cases, bypassing the complex step of re‑training from scratch. The platform further supports manual checks that flag broken links or outdated dependencies, ensuring that the catalog remains functional over time.

The project overcomes two principal obstacles that have limited the use of published AI models [16]. First, by summing up all eligible publications in medical literature, but also computer science journals and conference proceedings, extracting key metadata, and indexing the resulting model records in a faceted web interface, the platform unifies previously scattered code repositories and papers into a single searchable resource. In addition, every model record follows a unified schema that captures modality, subspecialty, intended use, data provenance, performance metrics and regulatory details, thereby providing a machine-readable description that facilitates automated analysis and evaluation. The dataset was further enriched with information that is often not present in the original publications, through an automated pipeline that interrogated each corresponding GitHub repository to verify the existence of pretrained model files and, when available, live web demonstrations.

The quantitative analyses of the extracted data suggest that publication output remains heavily concentrated in China and the United States, which coincides with rapid technological advances in AI in those geographical areas [17]. Architectural preferences have shifted toward CNNs and transformer‑based models. Modality-specialty pairings reveal a dominant focus on using MRI data for neuroradiology AI applications ( > 600 studies), followed by models for chest CT and X-ray, which confirms previous analyses [18]. On the contrary, nuclear‑medicine and positron emission tomography applications are scarce. In the word‑cloud analysis of reported limitations, the frequency of the keyword “external validation”, highlights a critical gap in robust model validation that could compromise generalizability.

Compared with existing resources, our work differs in scale and range of extracted information. The “MedicalModelLibrary” provides a manually curated list of models [12] but is limited to a few entries and focuses mostly on LLMs and Vision models. The RSNA ATLAS contains high‑quality, expert‑curated cards [11], but its coverage is also smaller than the present collection, and it does not contain a systematic analysis of code repositories. The “Awesome‑AI‑LLMs‑in‑Radiology” list aggregates only LLM applications [13], but does not deliver structured model records nor any health‑check of the associated repositories. By contrast, our platform integrates a searchable interface, expert curation and model records enriched with data from GitHub analysis, delivering a comprehensive, up-to-date resource for the community.

The study has several limitations. Firstly, model records were generated automatically with an open‑source LLM, and although stability and fidelity were quantified and all the records were manually reviewed by experts, the process can still produce omissions or minor inaccuracies, especially in cases where the original publication is locked or otherwise inaccessible. Users are therefore encouraged to flag in the repository incomplete or erroneous entries, and future releases will incorporate the human‑curated corrections. Flagged models are isolated and reviewed by the database curators prior to going live again in an updated version. Secondly, the underlying literature search was restricted to PubMed, arXiv, and Scopus using queries defined in Supplementary Table 1 to ensure reproducibility. While these queries were designed to be comprehensive, they may not have captured every available model in the literature, and users are therefore encouraged to enrich the repository through new submissions. Finally, due to computational constraints, the initial extraction did not populate every field mandated by the RSNA Roadmap, focusing more on correct indexing and access to manuscript and code.

In conclusion, OpenRad delivers a large-scale, curated repository of more than 1600 radiology AI models that are indexed, standardized, and enriched with verified information on code availability, pretrained weights, and live demos. It thus aims to resolve the longstanding problems of fragmented distribution and non-standard reporting, accelerate model discovery and reproducibility, while providing a transparent foundation for future benchmarking and clinical translation of AI in radiology.

## Supplementary information

ELECTRONIC SUPPLEMENTARY MATERIAL

## Abbreviations

AI: Artificial intelligence
AUC: Area under the curve
CNN: Convolutional neural network
CT: Computed tomography
DOI: Digital object identifier
DSC: Dice similarity coefficient
IRB: Institutional Review Board
JSON: JavaScript Object Notation
LLM: Large language model
MRI: Magnetic resonance imaging
OI: Oncological imaging
PSNR: Peak signal-to-noise ratio
RSNA: Radiological Society of North America
SSIM: Structural similarity index measure
