# Supplementary material for: OpenRad: a curated repository of open-access AI models for radiology
Source: Eur Radiol. 2026 May 28;36(10):7502–11. doi: 10.1007/s00330-026-12650-0 (PMC13574895; doi:10.1007/s00330-026-12650-0)
Supplement: Supplementary file 1 [file 330_2026_12650_MOESM1_ESM.pdf]

ELECTRONIC SUPPLEMENTARY MATERIAL

OpenRad: a Curated Repository of Open-access AI models for Radiology

Eur Radiol (2026) Vrettos K, Papadaki G, Brilakis E et al.

Database	Query
PubMed	("radiology"[Title/Abstract] OR "imaging"[Title/Abstract]) AND ("github"[Title/Abstract] OR "open access"[Title/Abstract]) AND ("AI"[Title/Abstract] OR "artificial intelligence"[Title/Abstract] OR "ML"[Title/Abstract] OR "machine learning"[Title/Abstract] OR "model"[Title/Abstract])
arXiv	(radiology OR imaging) AND (github OR "open access") AND (AI OR "artificial intelligence" OR ML OR "machine learning" OR model) AND submittedDate:[201512080000 TO 202512072359]
Scopus	(TITLE-ABS("radiology" OR "imaging") AND TITLE-ABS("github" OR "open access") AND TITLE-ABS("AI" OR "artificial intelligence" OR "ML" OR "machine learning" OR "model")) AND PUBYEAR > 2014

Supplementary Table 1: *Queries used to create the dataset*

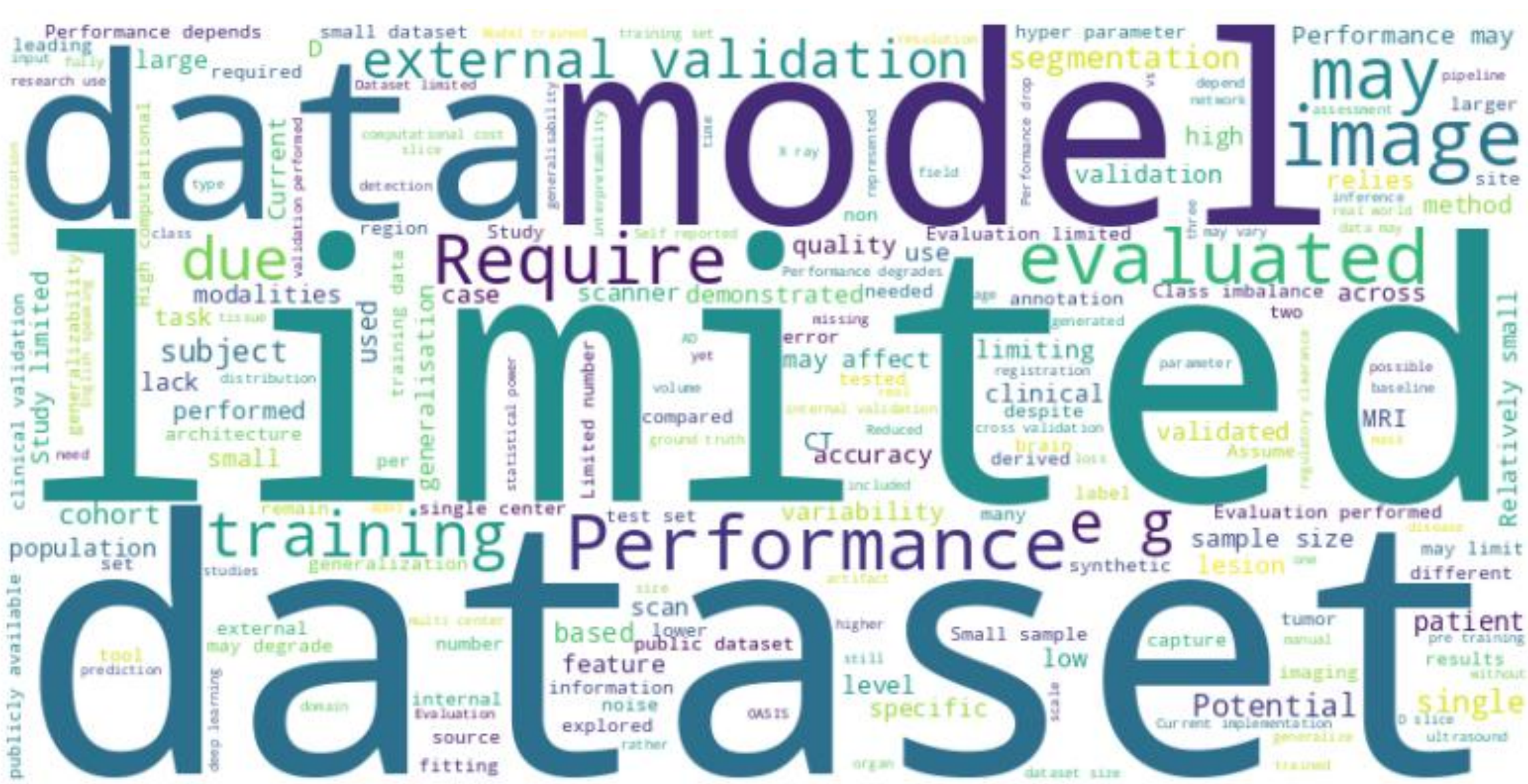

Supplementary Figure 1: *Wordcloud of most common words found in limitation sections*
